# Lung Ultrasound Eight-Point Method in Diagnosing Acute Heart Failure in Emergency Patients with Acute Dyspnea: Diagnostic Accuracy and 72 h Monitoring

**DOI:** 10.3390/medicina56080379

**Published:** 2020-07-28

**Authors:** Erika Glöckner, Felicitas Wening, Michael Christ, Alexander Dechêne, Katrin Singler

**Affiliations:** 1 Department of Gastroenterology, Hepatology, Endocrinology, Diabetology and Nutrition, Klinikum Nuernberg, Paracelsus Medical University Nuernberg, 90419 Nuernberg, Germany; alexander.dechene@klinikum-nuernberg.de; 2 Department of Respiratory Medicine, Allergology and Sleep Medicine, Klinikum Nürnberg, Paracelsus Medical University Nuernberg, 90419 Nuernberg, Germany; f.wening@gmx.de; 3 Emergency Department, Luzerner Kantonsspital, 6000 Luzern, Switzerland; michael.christ@luks.ch; 4 Department of Geriatrics, Klinikum Nuernberg, Paracelsus Medical University, 90419 Nuernberg, Germany; katrin.singler@klinikum-nuernberg.de; 5 Institute for Biomedicine of Aging, Friedrich-Alexander University Erlangen-Nuernberg, 90419 Nuernberg, Germany

**Keywords:** lung ultrasound, thoracic ultrasound, emergency department, emergency care, B-lines, B-line ultrasound, dyspnea

## Abstract

*Background and Objectives*: Acute dyspnea is a common chief complaint in the emergency department (ED), with acute heart failure (AHF) as a frequent underlying disease. Early diagnosis and rapid therapy are highly recommended by international guidelines. This study evaluates the accuracy of point-of-care B-line lung ultrasound in diagnosing AHF and monitoring the therapeutic success of heart failure patients. *Materials and Methods*: This is a prospective mono-center study in adult patients presenting with undifferentiated acute dyspnea to a German ED. An eight-zone pulmonary ultrasound was performed by experienced sonographers in the ED and 24 and 72 h after. Along with the lung ultrasound evaluation patients were asked to assess the severity of shortness of breath on a numeric rating scale. The treating ED physicians were asked to assess the probability of AHF as the underlying cause. Final diagnosis was adjudicated by two independent experts. Follow-up was done after 30 and 180 days. *Results*: In total, 102 patients were enrolled. Of them, 89 patients received lung ultrasound evaluation in the ED. The sensitivity of lung ultrasound evaluation in ED in diagnosing AHF was 54.2%, specificity 97.6%. As much as 96.3% of patients with a positive LUS test result for AHF in ED actually suffered from AHF. Excluding diuretically pretreated patients, sensitivity of LUS increased to 75% in ED. Differences in the sum of B-lines between admission time point, 24 and 72 h were not statistically significant. There were no statistically significant differences in the subjectively assessed severity of dyspnea between AHF patients and those with other causes of dyspnea. Of the 89 patients, 48 patients received the final adjudicated diagnosis of AHF. ED physicians assessed the probability of AHF in patients with a final diagnosis of AHF as 70%. Roughly a quarter (23.9%) of the overall cohort patients were rehospitalized within 30 days after admission, 38.6% within 180 days of follow-up. *Conclusion*: In conclusion, point-of-care lung ultrasound is a helpful tool for the early rule-in of acute heart failure in ED but only partially suitable for exclusion. Of note, the present study shows no significant changes in the number of B-lines after 24 and 72 h.

## 1. Introduction

Acute dyspnea is a common chief complaint in patients presenting to the emergency department (ED) and is associated with high morbidity and mortality. About 50% of adult dyspnoeic patients display acute heart failure (AHF) [1]. According to the setting prevalence of AHF ranges from 29% to 79% [2]. Rapid accurate diagnosis of AHF is hindered due to lacking sensitivity and specificity of patient history and the ambiguity of clinical signs and symptoms.

NT-proBNP is known to be a helpful biomarker to rule out AHF [3] but is not quickly available in all hospital settings. Lung ultrasound (LUS) is a non-invasive tool for the evaluation of patients with shortness of breath. Point of care ultrasound is a fast method of bedside evaluation of patients in the emergency room and suitable for the examination of patients with acute shortness of breath. Ultrasound can, therefore, be used as an extension of the physical examination. Furthermore, no direct costs incur and the technique is both easy-to-teach and easy-to-use. Different LUS methods have been evaluated (eight-zone technique, lung comet score, combination of lung and cardiac ultrasound including ejection fraction [EF]). We considered the eight-zone technique to be the most practicable method in the emergency department setting and recommended in the consensus guidelines [4].

The primary aim of this pilot study was to determine the diagnostic accuracy of point of care ultrasound in diagnosing AHF as underlying disease of acute dyspnea in the ED, while the secondary aim was to evaluate if lung ultrasound (LUS) is a suitable tool for monitoring therapeutic outcomes after 24 and 72 h in hospitalized patients.

## 2. Materials and Methods

### 2.1. Study Design and Patients

This is a prospective monocentric observational pilot study in adult patients presenting with undifferentiated acute dyspnea to the emergency department of a German university hospital with over 60,000 annual ED visits. The study was conducted according to the principles of good clinical practice and the Declaration of Helsinki in its latest version and was positively evaluated by the Institutional Review Board of the Friedrich-Alexander University Erlangen-Nuernberg (Re.-No. 72_13 B). Patients were admitted between August 2014 and May 2015 as sub-study patients within the “Registry of Patients with Acute Dyspnea in the Emergency Department” project, registered at clinicalstrials.gov (NCT01910233). An interim analysis of the first enrolled 25 patients has been published previously [5].

Inclusion criteria were: ≥18 years, and a chief complaint of undifferentiated acute shortness of breath.

Exclusion criteria: pleural effusion in more than two quadrants, known pneumothorax, lack of lung sliding, interstitial lung disease, radiologically-confirmed pneumonia, status after lung resection, known lung cancer, mechanical ventilation or presence of pleural drainage.

**Figure 1:**
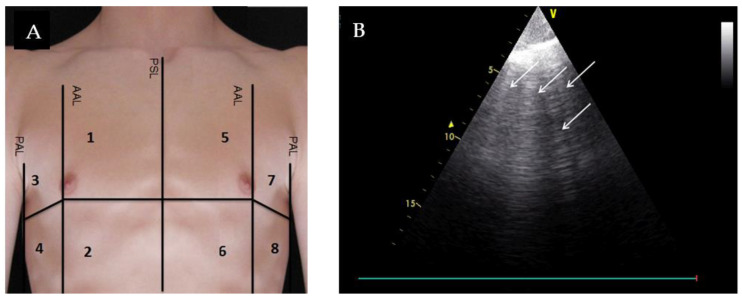
(**A**) Division of the thorax in eight lung zones. PSL: parasternal line; AAL: anterior axillary line; PAL: posterior axillary line; (**B**) Example of a lung ultrasound loop of quadrant 1 at admission assessed as a positive region due to the appearance of ≥3 B-lines.

After obtaining written informed consent patients received an eight-zone LUS evaluation [4], four zones for each hemi-thorax (2–5 MHz phased array transducer, General Electric Vivid S6, probe position transversal to the ribs with an imaging depth of 18 cm) in addition to routine diagnostics. During their stay in the emergency department LUS loops were recorded by two student sonographers within one hour after the admission to the ED, especially trained in the LUS technique by two experts in internal and emergency medicine. LUS evaluation was performed in patients in a supine position, screening eight quadrants of the thorax, in ascending order with a six second clip length. Figure 1A shows the division of the thorax in eight lung zones. For each quadrant one ultrasound loop was saved pseudonymized on a digital medium and afterwards evaluated independently by two expert emergency sonographers in a standardized manner, blinded to clinical data (Cohen’s Kappa = 0.9).

LUS result was positive for AHF if LUS showed a bilateral existence of two or more regions with three or more B-lines [5], for example ≥3 B-lines in quadrant one, four, five, and seven. Figure 1B shows an example of a positive quadrant with ≥3 B-lines. To follow up the exertion of the B-lines after initial treatment, lung ultrasound evaluation was repeated after 24 and 72 h, provided the patient was not yet discharged and still available for follow up measurements on the ward. Along the ultrasound evaluation time points NT-proBNP was measured on admission day, and after 24 and 72 h. Additionally, patients were asked to assess their shortness of breath on a numeric rating scale from 0 to 10, where 0 corresponds to ‘no dyspnea’ and 10 to the ‘strongest dyspnea one can imagine’. Furthermore, the attending emergency physician was asked to assess the probability of AHF as underlying cause of dyspnea on a scale of 0 (impossible) to 10 (very likely) after completion of medical history and clinical examination.

In addition to ultrasound and demographic data, data on comorbidities, diagnostics, and therapies were collected.

In order to assess the diagnostic efficacy of LUS in the diagnosis of acute heart failure, a final adjudicated diagnosis of acute heart failure was conducted by two experienced physicians (expert in cardiology, and an emergency physician) taking all routine medical record data into account. In the case of a disagreement about AHF diagnosis, a third investigator’s judgment was included [6,7]. The ‘final adjudication data entry sheet’ and ‘manual’ are shown in the appendix. Primary endpoint was the diagnostic accuracy of LUS in the diagnosis of AHF. The secondary aim was to evaluate if LUS was a suitable tool for the monitoring of therapy success after 24 and 72 h. After 30 and 180 days the patients were followed up by telephone contact for survival status. If a patient could not be reached by phone, he was contacted via mail, family doctor, or registration office request.

### 2.2. Statistics

Continuous variables are represented as means (± SD) or medians (interquartile range (IQR)), categorical variables as numbers and percentages. Comparisons in different subgroups of patients were performed using the Mann–Whitney U test for independent variables. For categorical data the Pearson chi-square, respectively Fisher’s exact test was used. The Wilcoxon test was used to calculate differences in a group of changing diagnostic parameters over time. Proportions are described with 95% confidence intervals (CI). *p*-values < 0.05 were considered statistically significant. Data were analyzed using SPSS IBM Statistics 23 version for Windows (Munich, Germany).

## 3. Results

**Figure 2:**
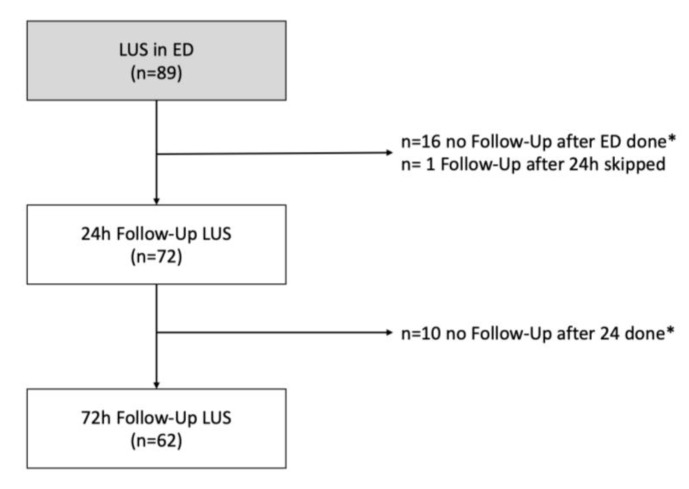
Patients with lung ultrasound measurement in the ED, 24 h, and 72 h after presentation. LUS: Lung Ultrasound, * LUS was not performed because the patient had already been discharged or the ultrasound device was not ready for use due to technical reasons or the patient was not present at the agreed time point for follow-up measurements on the ward.

A total of 102 patients were enrolled; 89 of 102 patients received lung ultrasound evaluation in the emergency department (Figure 2).

Median age of patients who received LUS measurement in ED was 73 years, 62% of them had a previous history of heart failure and 71% of hypertension (Table 1). 48 of 89 patients received the final adjudicated diagnosis of acute heart failure (AHF), 41 patients suffered from other diseases (no AHF). AHF patients were older (76 vs. 63 years, *p* < 0.001), suffered more often from hypertension (95.8% vs. 61%, *p* < 0.001) and oxygen saturation was lower than in patients with other diseases; whereas creatinine, baseline NT-proBNP, and baseline Troponin T was higher (Table 2).

Subjectively-assessed severity of dyspnea of patients with LUS examination in the ED was 8 at the time point of arrival of the EMS team and reduced to 5 in the emergency department, and 3 after 24 and 72 h (Table 2). There was no statistically significant difference in the subjectively-assessed severity of dyspnea between the two groups AHF and no AHF.

Probability for AHF was assessed at 70% in patients with an adjudicated final diagnosis of AHF and at 34% in patients with an adjudicated final diagnosis of no AHF by the attending emergency physician.

### 3.1. Diagnostic Accuracy

A total of 54.2% of AHF patients showed a positive LUS test result for acute heart failure at presentation, 18.2% after 24 h and 19.5% after 72 h. Patients with a positive LUS test in the ED had in total a median of 19 B-lines, IQR (16–23). Of those patients with a LUS evaluation in the ED, 18% have been pretreated with diuretics by the admitting family physician or the emergency doctor (33.3% (AHF) vs. 0% (no AHF), *p* < 0.001), Table 3.

Diagnostic accuracy is shown in Table 4. Sensitivity: In 54.2% of the recorded loops of patients with a final adjudicated diagnosis of AHF showed a bilateral existence of two or more positive regions with three or more B-lines and therefore indicated AHF. Specificity: 97.6% of patients with a final adjudicated diagnosis of no AHF had a negative LUS test result.

Positive predictive value: 96.3% of patients with a positive LUS test result for AHF actually suffered from AHF according to the adjudicated final diagnosis. Negative predictive value: 64.5% of patients with a negative LUS test result actually suffered from other diseases than AHF according to the adjudicated final diagnosis.

**Figure 3:**
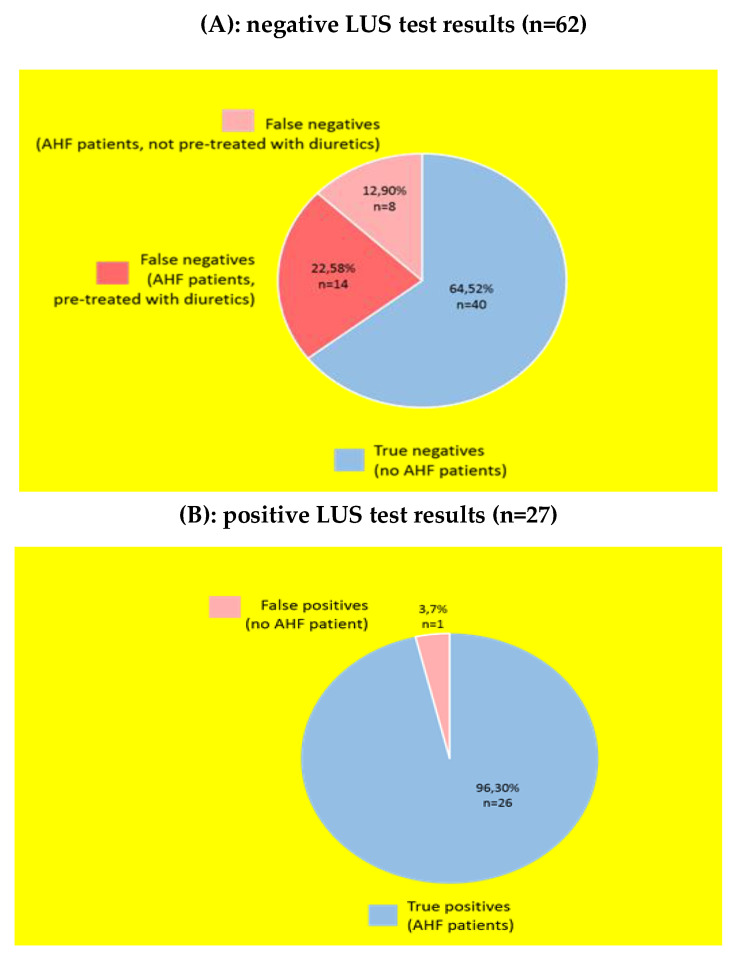
Amount of (**A**) true/false negative and (**B**) true/false positive test results among acute dyspnea patients.

Figure 3 visualize the amount of true and false negative/positive test results. Figure 3A emphasizes the issue of false negative test results among patients with an adjudicated final diagnosis of AHF. In 22 of 62 patients with a negative LUS test result suffered from AHF as underlying disease of their dyspnea, although the LUS technique did not indicate AHF. Fourteen of 22 patients were already pretreated with diuretics by their admitting family physician or emergency doctor. Figure 3B visualizes the high positive predictive value of 96.3%.

In a sensitivity analysis, we examined the cohort of patients not diuretically pretreated in the prehospital scene (Table 5): we found a higher sensitivity (75% vs. 54.2% diuretically pretreated) and negative predictive value (83.3% vs. 64.5% diuretically pretreated).

### 3.2. LUS for Therapeutic Monitoring

**Figure 4:**
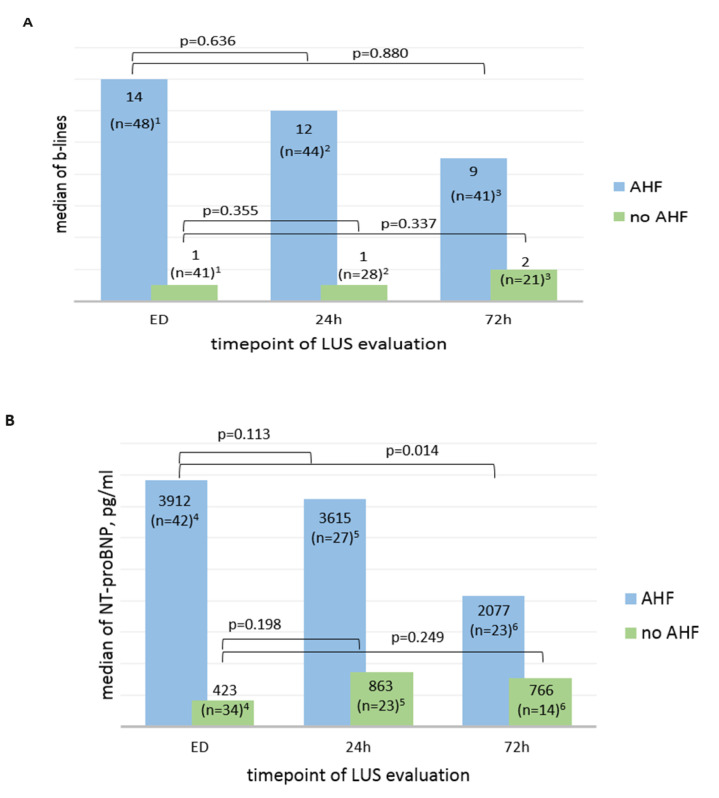
Changes in median B-lines (**A**) and median NT-proBNP (**B**) in ED, 24 h, and 72 h after admission, divided by the final adjudicated diagnosis “AHF” and “no AHF”. (**A**) ^1^ number of patients who received LUS in ED; ^2^ number of patients who received LUS in ED and after 24 h; ^3^ number of patients who received LUS in ED and after 72 h. (**B**) ^4^ number of patients who received NT-proBNP in ED; ^5^ number of patients who received NT-proBNP in ED and after 24 h; ^6^ number of patients who received NT-proBNP in ED and after 72 h.

Figure 4 summarizes B-lines (A) and NT-proBNP (B) at admission, and after 24 and 72 h for ‘AHF’ and ‘no AHF’ patients. The median of B-lines of AHF patients decreased over time from initially from 14 to 12 after 24 h (*p* = 0.636) and to 9 after 72 h (*p* = 0.880), NT-proBNP also from 3912 pg/mL to 3615 pg/mL after 24 h (*p* = 0.113) and 2077 pg/mL after 72 h (*p* = 0.014). However, in patients with other diagnoses than AHF, the median of B-lines and NT-proBNP increased slightly over time.

**Figure 5:**
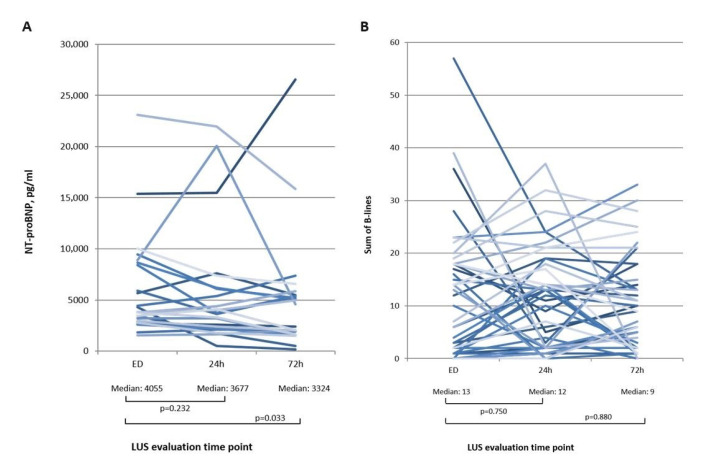
(**A**) Individual dynamic NT-proBNP changes. (**B**) Individual dynamic sum of B-line.

Figure 5A visualizes the NT-proBNP development within hospitalization for each individual AHF patient with a complete NT-proBNP follow up (*n* = 20). NT-proBNP values after 72 h were significantly lower than at the time point of admission (asymptomatic Wilcoxon test: z: −2.128, *p* = 0.033, *n* = 20). Figure 5B shows the individual sums of B-lines over time of 41 AHF patients with a complete LUS follow-up. According to the *p*-values measured via the Wilcoxon test, differences in the sum of B-lines between the admission time point, 24 h, and 72 h were not statistically significant.

**Figure 6:**
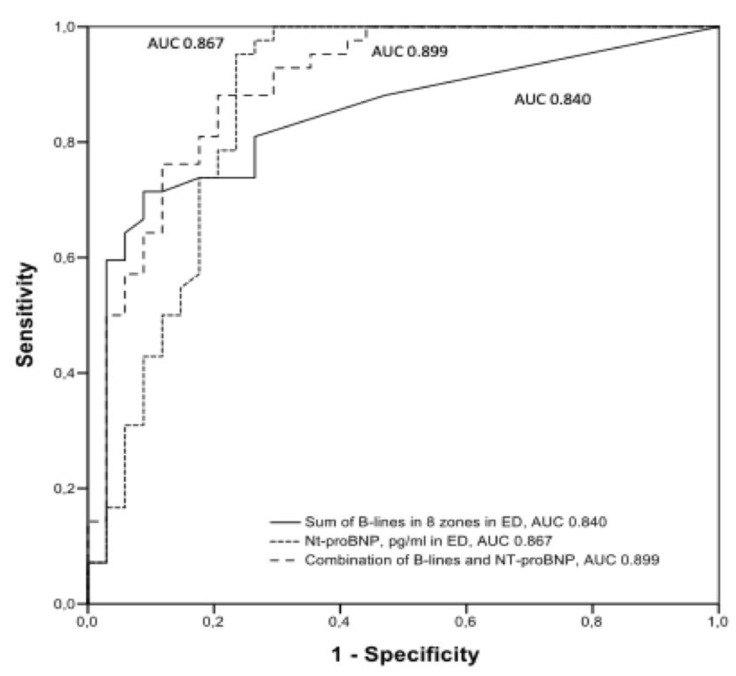
Area under the receiver characteristic operating curve (AUC) of the sum of B-lines, NT-proBNP, and a combination of both diagnostic tests.

The area under the receiver characteristic operating curve (AUC) of the sum of B-lines in eight zones in the ED was 0.840 and of NT-proBNP 0.867, respectively. The combination of B-lines and NT-proBNP achieved an AUC of 0.899 (see Figure 6).

### 3.3. Mortality and Rehospitalization of Patients with Acute Dyspnea

Table 6 shows the length of stay, hospital mortality, and survival after six months. The length of stay of AHF patients was significantly longer than length of stay of no AHF patients (eight days vs. four days). In-hospital mortality of acute dyspnea patients that underwent LUS examination was 1.1% (*n* = 1). After six months 83.3% of AHF patients were known to be alive, 6.3% were deceased and 10.4% were lost to follow up with an unknown survival status. There is no statistically significant difference regarding the survival status between the groups ‘AHF’ and ‘no AHF’. A total of 23.9% of the overall cohort were rehospitalized within 30 days after admission, 38.6% within 180 days of follow up.

## 4. Discussion

B-line LUS is proposed as a tool used at the point of care to support clinical decision-making in patients with acute dyspnea [4,8,9,10,11]. In our study visualizing three or more B-lines in two or more zones of each hemithorax showed very high specificity (97.6%) and moderate sensitivity (54.2%) in the diagnosis of AHF [9,11].

Pivetta et al. analyzed B-lines with high diagnostic accuracy for AHF (SE 97%, SP 97.4%) using a method with six scans [12]. The current study extends previous methods using eight scans in the ED as recommended in a consensus statement [4,13]. With the aim of identifying the most correct number of B-lines as possible, a clip length of six seconds was chosen [13]. It is possible that the number of B-lines was underestimated, as in the present study a display length of 18 cm was used, whereas B-lines, by definition, have to reach the bottom of the LUS -loop. Other studies also used shorter display lengths [14]. Anderson et al. demonstrated, that using LUS (probe was placed perpendicular to the rips with the maximum screen deep of 15 cm) a sensitivity of 70% and a specificity of 75% could be achieved to detect AHF [15]. 

There are different methods for the quantification of B-lines [14]. We decided to count the highest number of B-lines during the runtime of the recording, as in our opinion this is the most useful method to identify ED patients with AHF using LUS. As there exists no standardization in the evaluation of B-lines, it seems reasonable to hypothesize that the wide range of sensitivity (58−92%) and specificity (75–100%) is affected by the method used [15,16,17,18,19].

There are several possible explanations for the moderate sensitivity of our study findings compared to others.

Our cohort showed a median age of 73 years and was therefore older than cohorts of comparable studies (73 years vs. median age of 64 [18] and 53 [20]). In contrast to other ED studies, almost all of our AHF patients had a history of CHF (97.9% compared to 75% described by Anderson [15]). Furthermore, there are notable differences in the inclusion criteria compared to other studies as patients with renal dysfunction and preexisting lung diseases were not excluded [18,21,22]. Higher age is associated with an increased number of comorbidities and might, therefore, hinder the visibility of B-lines causing a lower sensitivity. Of note, 18% of our patients have been pretreated with diuretics in an outpatient facility or by paramedics and family doctors, before admission to the hospital. Fourteen of 22 AHF patients, who could not be detected via LUS (false negatives), actually received a diuretic pretreatment. Excluding those pretreated patients from analysis, the sensitivity for the detection of AHF with LUS increased up to 75% while the specificity remained unchanged (97.6%).

Volpicelli et al. conducted a study to monitor the number of B-lines in patients with heart failure. After applying diuretic treatment, they found a significant decrease of B-lines [21]. LUS in our ED was performed within 1 h after the admission to the ED department. B-lines might already have been reduced by the prehospital treatment with diuretics. The study of Prosen et al. describes a 100% sensitivity of LUS in detecting AHF in patients with dyspnea before the application of medication in a prehospital emergency setting [23]. Sartini et al. stated that patients who were not diuretically pretreated performed better in terms of detecting AHF by LUS [19]. Therefore, we assume that, in our study, pretreatment of patients with severe heart failure (NYHA ≥ 3) resulted in a lower sensitivity of the LUS test.

Final adjudicated diagnosis of AHF was determined by two experienced physicians (cardiologist, emergency physician) using all available patient data for adjudication [1,24,25].

As there exists no consistent gold standard in the determination of heart failure, we based our assessment on the BASEL study. Possibly, we therefore identified a higher rate of AHF patients compared to other studies [1,23].

The number of B-lines is assumed to decrease with treatment for AHF. Therefore, we repeated LUS after 24 and 72 h, in order to monitor the therapeutic outcome in hospitalized patients.

Against all expectations, the present study showed no significant changes in the number of B-lines in our sample of 41 AHF patients with complete follow up after 24 h and 72 h (median of 13 B-lines in the ED vs. nine B-lines after 72 h, *p* = 0.880). In contrast to our findings, Gargani et al. showed a significant reduction in B-lines from 48 ± 48 B-lines at admission to 20 ± 23 at the time point of discharge (*p* < 0.001) [26]. It might have been advantageous to monitor B-lines just prior to discharge, however, we decided to choose a precise follow up time point in favor of standardization. In our cohort, the length of stay of AHF patients was quite different (eight days with an interquartile range of 5–12). However, Spevack et al. also did not find a statistical significance in monitoring B-lines in a study of 50 AHF patients after 24 h and before discharge [22].

In our cohort median of NT-pro BNP (high diagnostic accuracy for diagnosis of AHF [27,28]) is still high after 72 h, although it shows a statistically significant decrease after 72 h in AHF patients (3912 pg/mL vs. 2077 pg/mL, *p* = 0.014). Even the subjectively assessed dyspnea of AHF patients is still 3 (median) on a scale from 0 to 10, compared to 5 (median) at admission. One assumption is that older patients, as in our study, suffering from severe AHF with NYHA ≥ 3, need more time for symptom relief than younger ones. Therefore, B-lines might not have yet attained a statistically significant decrease within 72 h of the follow-up time.

There are several limitations in our study. It is a small single center study examining 102 patients. Additionally, there was a high dropout rate of patients not completing all LUS tests in ED, after 24 and 72 h (32.2%), due to organizational or logistic issues. However, data were obtained prospectively, B-lines were checked and counted by blinded expert sonographers suggesting valid data. We included a high amount of diuretically pretreated AHF patients limiting the diagnostic accuracy of LUS in our analysis. In this patients LUS was not a supportive tool in detecting the AHF diagnosis, leading to false negative test results and a moderate negative predictive value.

## 5. Conclusions

According to our data, LUS is a helpful tool for the early rule-in of acute heart failure in ED but only partly suitable for exclusion.

Considering the fact that the majority of patients, in whom LUS could not detect AHF, were pretreated with diuretics, it is tempting to speculate that LUS reacts very fast to changes of intrapulmonary congestion. Therefore, a combined strategy of NT-ProBNP testing and LUS will lead to fast and reliable results to support the diagnosis of AHF.

It can be assumed that ultrasound monitoring could provide relevant results after more than 72 h, but the present study does not show significant changes in the number of B-lines after 24 and 72 h.

## Figures and Tables

**Table 1:** Demographics of eligible patients with acute dyspnea in the emergency department, divided by their final adjudicated diagnosis “AHF” and “no AHF”.

	Overall Cohort (*n* = 89)	AHF (*n* = 48)	No AHF (*n* = 41)	*p*
Age, Median (IQR), Years	73 (60–80)	76 (70–81)	63(50–75)	**<0.001**
Males, *n* (%)	52 (58.4)	28 (58.3)	24 (58.5)	0.985
BMI, Median (IQR), kg/m^2^	28 (25–32)	28 (26–33)	28 (25–31)	0.545
Care Home Resident, *n* (%)	7 (7.9)	7 (14.6)	0 (0)	**0.011**
NYHA ≥ III, *n* (%), *n* = 72	59 (81.9)	38 (95.0)	21 (65.6)	**0.001**
**Relevant Comorbidities, *n* (%)**				
Myocardial Infarction	9 (10.1)	7 (14.6)	2 (4.9)	0.130
Chronic Heart Failure	62 (69.7)	47 (97.9)	15 (36.6)	**<0.001**
Peripheral Vascular Disease	7 (7.9)	2 (4.2)	5 (12.2)	0.161
Chronic Lung Disease	25 (28.1)	12 (25)	13 (31.7)	0.483
Cerebrovascular Disease	9 (10.1)	5 (10.4)	4 (9.8)	0.918
Diabetes Mellitus	29 (32.6)	22 (45.8)	7 (17.1)	**0.005**
Moderate and Severe Kidney Disease	33 (37.1)	25 (52.1)	8 (19.5)	**0.002**
Solid Tumor	14 (15.7)	7 (14.6)	7 (17.1)	0.748
**CCI Overall Score, Median (IQR)**	3 (1–5)	4 (2–6)	2 (0–4)	**0.002**
**Cardiovascular Risk Factors, *n* (%)**				
Arterial Hypertension	71 (79.8)	46 (95.8)	25 (61)	**<0.001**
(Ex-)smoker	55 (64)	28 (59.6)	27 (69.2)	0.353
Hyperlipidemia	10 (11.2)	8 (16.7)	2 (4.9)	0.079
Hypercholesterolemia	15 (16.9)	9 (18.8)	6 (14.6)	0.605
Obesity	49 (55.1)	25 (52.1)	24 (58.5)	0.542
Positive family history	10 (11.2)	4 (8.3)	6 (14.6)	0.348

**Table 2:** Admission related information of eligible patients with acute dyspnea in the emergency department, divided by their final adjudicated diagnosis “AHF” and “no AHF”.

	Overall Cohort (*n* = 89)	AHF (*n* = 48)	No AHF (*n* = 41)	*p*
**Admission, *n* (%)**				
Emergency Service with Doctor	8 (9.0)	5 (10.4)	3 (7.3)	0.610
Emergency Service	6 (6.7)	2 (4.2)	4 (9.8)	0.295
Self Admission	16 (18.0)	5 (10.4)	11 (26.8)	0.044
Family Physician	55 (61.8)	33 (68.8)	22 (53.7)	0.144
Rehabilitation	4 (4.5)	3 (6.3)	1 (2.4)	0.387
**Vital signs in ED, Median (IQR)**				
Respiratory Rate, breaths per minute	17 (14–20)	18 (15–20)	16 (14–20)	0.373
Systolic Blood Pressure, mmHg	136 (121–150)	138 (122–151)	133 (120–150)	0.529
Heart Rate, beats per minute	86 (74–100)	80 (71–94)	89 (80–108)	0.022
Oxygen Saturation, %	96 (93–98)	95 (92–98)	97 (94–99)	0.037
**Lab, Median (IQR)**				
Potassium, mmol/L	4.2 (3.9–4.5)	4.3 (3.9–4.7)	4.2 (3.9–4.4)	0.496
Sodium, mmol/L	140 (137–142)	141 (137–143)	140 (138–142)	0.791
Creatinine, mg/dL	1.06 (0.87–1.51)	1.24 (0.98–1.77)	0.93 (0.77–1.18)	<0.001
Urea, mg/dL	36 (27–57)	52 (35–74)	29 (23–36)	<0.001
Hemoglobin, g/dL	13.2 (11.6–14.6)	12.4 (11.0–14.1)	14.1 (12.7–15.1)	0.003
Leukocytes/nL	8.7 (7.2–11.0)	8.5 (7.3–10.5)	9.2 (6.8–12.0)	0.301
Glucose, mg/dL	123 (105–147)	126 (103–170)	122 (107–138)	0.385
NT-proBNP, pg/mL	2648 (763–5798)	3912 (2594–8855)	423 (63–1325)	<0.001
hs cTnT, ng/L	21 (14–42)	31 (14–48)	14 (14–26)	0.010
**Severity of dyspnea on a numeric rating scale (1–10), Median (IQR)**				
At Arrival of Emergency Service (*n* = 19)	8 (4–9)	8 (6–10)	4 (3–7)	0.170
At Admission (*n* = 79)	5 (2–6)	5 (3–6)	3 (1–6)	0.071
At 24 h (*n* = 70)	3 (1–5)	4 (2–5)	2 (0–5)	0.042
At 72 h (*n* = 57)	3 (1–4)	3 (1–4)	2 (0–5)	0.542

**Table 3:** Lung ultrasound findings and diuretic-treatment status in ED.

	(*n* = 89)	AHF (*n* = 48)	No AHF (*n* = 41)	*p*
**Positive LUS for acute heart failure, *n* (%)**				
in ED	27 (30.3)	26 (54.2)	1 (2.4)	**<0.001**
After 24 h	11 (15.3) (*n* = 72) ^1^	8 (18.2) (*n* = 44) ^1^	3 (10.7) (*n* = 28) ^1^	0.391
After 72 h	11 (17.7) (*n* = 62) ^1^	8 (19.5) (*n* = 41) ^1^	3 (14.3) (*n* = 21) ^1^	0.610
**Pretreatment with diuretics, *n* (%)**	16 (18)	16 (33.3)	0 (0)	**<0.001**

IQR: Interquartile Range, BMI: Body Mass Index, NYHA: New York Heart Association, CCI: Charlson’s Comorbidity Index, ED: Emergency Department, LUS: Lung Ultrasound, ^1^ Lower patient numbers according to Figure 2.

**Table 4:** Diagnostic accuracy of LUS in diagnosing AHF in dyspnea patients in ED.

SE (95% CI)	SP (95% CI)	PPV (95% CI)	NPV (95% CI)	LR+ (95% CI)	LR- (95% CI)
54.2 (39.2–68.6)	97.6 (87.1–99.9)	96.3 (78.7–99.5)	64.5 (57.1–71.3)	22.2 (3.2–156.6)	0.47 (0.34–0.64)

**Table 5:** Diagnostic accuracy of LUS in diagnosing AHF in non-pretreated dyspnea patients with diuretics in ED.

SE (95% CI)	SP (95% CI)	PPV (95% CI)	NPV (95% CI)	LR+ (95% CI)	LR- (95% CI)
75 (56.6–88.5)	97.6 (87.1–99.9)	96 (77.4–99.4)	83.3 (73.3–90.1)	30.8 (4.4–215.3)	0.26 (0.14–0.47)

**Table 6:** Length of stay, mortality of eligible patients within in-hospital stay and survival status 180 days after index hospitalization.

	Overall Cohort (*n* = 89)	AHF (*n* = 48)	No AHF (*n* = 41)	*p*
Length of Stay, Median (IQR)	7 (4–11)	8 (6–12)	4 (1–9)	**0.004**
Hospital Mortality, *n* (%)	1 (1.1)	0 (0)	1 (2.4)	0.277
Survival After 6 Months, *n* (%)	74 (83.1)	40 (83.3)	34 (82.9)	0.934
Dead After 6 Months, *n* (%)	5 (5.6)	3 (6.3)	2 (4.9)	0.779
Unknown Survival Status After 6 Months, *n* (%)	10 (11.2)	5 (10.4)	5 (12.2)	0.779
